# Factors Influencing Oral Bioavailability of Thai Mango Seed Kernel Extract and Its Key Phenolic Principles

**DOI:** 10.3390/molecules201219759

**Published:** 2015-11-30

**Authors:** Pimsumon Jiamboonsri, Pimolpan Pithayanukul, Rapepol Bavovada, Jiraporn Leanpolchareanchai, Taijun Yin, Song Gao, Ming Hu

**Affiliations:** 1 Department of Pharmacy, Faculty of Pharmacy, Mahidol University, 447 Sri-Ayuthaya Road, Rajathevi, Bangkok 10400, Thailand; jiamboonsri_p@hotmail.co.th (P.J.); jiraporn.lea@mahidol.ac.th (J.L.); 2 Department of Pharmacological and Pharmaceutical Sciences, College of Pharmacy, The University of Houston, 1441 Moursund Street, Houston, TX 77030, USA; taijunyin@hotmail.com (T.Y.); sgao3@uh.edu (S.G.); mhu@uh.edu (M.H.); 3 Department of Pharmacognosy and Pharmaceutical Botany, Faculty of Pharmaceutical Sciences, Chulalongkorn University, 254 Phayathai Road, Pathumwan, Bangkok 10330, Thailand; rapepol1@hotmail.com

**Keywords:** *Mangifera indica* L., pentagalloyl glucopyranose, pharmacokinetic, Caco-2 cells, fecal lysate

## Abstract

Mango seed kernel extract (MSKE) and its key components (gallic acid, GA; methyl gallate, MG; and pentagalloyl glucopyranose, PGG) have generated interest because of their pharmacological activities. To develop the potential use of the key components in MSKE as natural therapeutic agents, their pharmacokinetic data are necessary. Therefore, this study was performed to evaluate the factors affecting their oral bioavailability as pure compounds and as components in MSKE. The *in vitro* chemical stability, biological stability, and absorption were evaluated in Hanks’ Balanced Salt Solution, Caco-2 cell and rat fecal lysates, and the Caco-2 cell model, respectively. The *in vivo* oral pharmacokinetic behavior was elucidated in Sprague-Dawley rats. The key components were unstable under alkaline conditions and in Caco-2 cell lysates or rat fecal lysates. The absorptive permeability coefficient followed the order MG > GA > PGG. The *in vivo* results exhibited similar pharmacokinetic trends to the *in vitro* studies. Additionally, the co-components in MSKE may affect the pharmacokinetic behaviors of the key components in MSKE. In conclusion, chemical degradation under alkaline conditions, biological degradation by intestinal cell and colonic microflora enzymes, and low absorptive permeability could be important factors underlying the oral bioavailability of these polyphenols.

## 1. Introduction

Mango (*Mangifera indica* L.) belongs to the family *Anacardiaceae* and is one of the most popular edible fruits around the world because of its unique taste and nutritional value. Mango is an excellent source of antioxidants, including phenolic compounds, carotenoids, and vitamin C [1]. The seed kernels, which are considered waste or by-products, have been extensively studied for their phytochemical and pharmacological activities. In addition to fat, oils and starches, other phytochemical compounds such as tannins, coumarin, ellagic acid, vanillin, mangiferin, ferulic acid, and cinnamic acid have been observed and characterized in mango kernels [2,3]. In the Thai mango cultivar “Fahlun”, the ethanolic extract of the seed kernels contains a relatively high amount of pentagalloyl glucopyranose (PGG), and relatively smaller amounts of gallic acid (GA) and methyl gallate (MG) [4]. Mango seed kernel extract (MSKE) and its major phenolic constituents have been shown to exert interesting pharmacological activities both *in vitro* and *in vivo*, including *in vitro* anti-methicillin-resistant *Staphylococcus aureus* activity [5], potent free radical scavenging and antioxidant, anti-inflammatory [4], and anti-enzymatic (e.g., tyrosinase, phospholipase and hyaluronidase) activities [6,7,8] and *in vivo* anti-hepatotoxicity activity against liver damage induced by carbon tetrachloride [4].

To enhance the potential of MSKE and it key components as natural therapeutic agents for disease treatment, their pharmacokinetic characteristics must be understood. The oral pharmacokinetics of GA have been extensively studied in both animal and human models [9,10,11,12,13]. In rats, GA was rapidly absorbed with a time to reach maximum plasma concentration (*T*_max_) for approximately 60 min after oral administration (p.o.) of GA as a pure compound or as an active component of rhubarb and grape seed extract [9,10,11]. GA also demonstrated a low maximum plasma concentration (*C*_max_) of 0.71 µmol/L and a fast elimination haft-life (*t*_1/2_) of 24 min when GA was orally administrated as a pure compound at a dose of 100 µmol/kg in rats [9]. In humans, after the administration of a single oral dose of acidum gallicum tablets or tea (each containing 0.3 mmol GA), the *C*_max_ and *t*_1/2_ of GA was approximately 2 µmol/L and 1 h, respectively [12,13]. Whereas the oral pharmacokinetics of GA have been widely studied, the pharmacokinetic characteristics of MG and PGG have not been as well elucidated. When the mixture of MG and PGG was administrated via intraperitoneal (i.p.) injection in rats at a same dose of 20 mg/kg, the blood levels of MG and PGG were in the micromolar levels, with *C*_max_ of 35 and 6 µM, respectively [14]. However, the plasma PGG levels were below the limit of detection (*i.e*. sub-micromolar) even when orally administered at a high dose of 80 mg/kg PGG in mice [15]. The poor oral bioavailability of drugs, which are affected by the drug absorption factors, such as the physicochemical properties, biological stability, gut microflora transformation, intestinal permeation, and hepatic metabolism, lead to correspondingly low biological efficacies *in vivo* [16]. Therefore, it is necessary to investigate the potential factors responsible for the low systemic levels of these phenolic compounds. Additionally, the plant matrix or the other components present in extracts have been reported to alter the pharmacokinetics of the active components. The active phenolic compounds of hawthorn and green tea displayed different pharmacokinetic behaviors in rats when orally administrated as pure compounds compared with when orally administrated as the extract [17,18]. Therefore, the aims of this study were to investigate the *in vitro* chemical and biological stabilities and the *in vitro* transport using the Caco-2 cell model of the three phenolic principles as pure compounds and as principles in MSKE. In addition, the *in vivo* oral pharmacokinetic behaviors of MG and PGG as the mixture and the principles in MSKE were elucidated in Sprague-Dawley (SD) rats.

## 2. Results and Discussion

### 2.1. Chemical Stability in Hanks’ Balanced Salt Solution (HBSS) at pH 5, 6, 7 and 8

**Figure 1:**
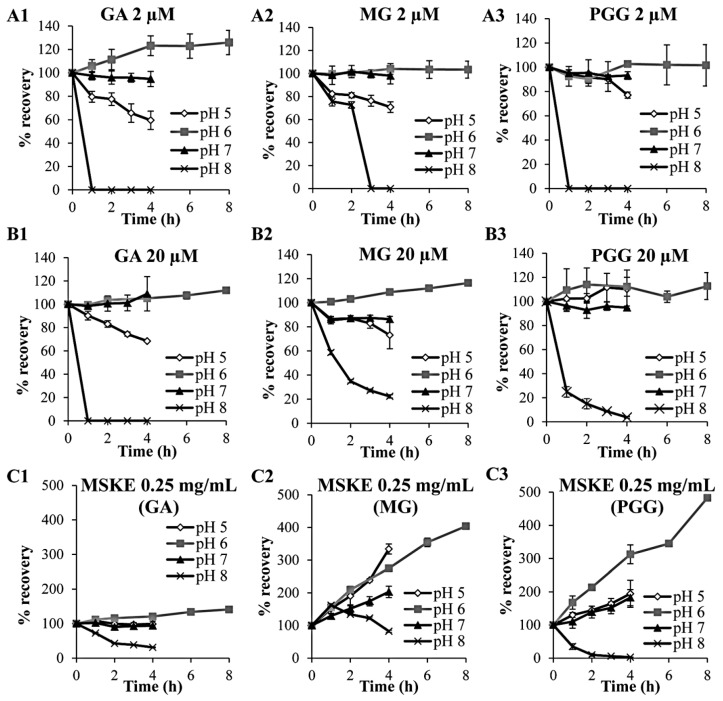
Chemical stabilities of GA, MG and PGG after incubation with HBSS at pH 5, 6, 7 and 8 as individual compounds (2 µM, **A**; and 20 µM, **B**) and as principles in MSKE (0.25 mg/mL, **C**). Each symbol indicates the mean ± S.D. (*n* = 3).

At concentrations of 2 and 20 µM, all three individual standard phenolic compounds decomposed in HBSS at pH 8 within 4 h, whereas they were stable in HBSS at pH 6 for 8 h (Figure 1A,B). After incubation in HBSS at pH 5 or 7 at low and high concentrations for 4 h, PGG was also stable, whereas GA and MG were only stable at pH 7. These results are consistent with the study of Friedman and Jürgens [19] who demonstrated that plant phenolic compounds were not stable in high pH environments in a time-dependent manner. Therefore, further experiments in this study were conducted at pH 6.

In contrast, regarding the MSKE principles, GA, MG and PGG demonstrated different characteristics after incubation at various pH conditions as compared with the pure compounds. Although, GA and PGG were similarly decomposed at pH 8 within 4 h, MG exhibited a slightly decreased recovery after incubation in HBSS at pH 8 for 4 h. After incubation in HBSS at pH 5, 6, or 7, GA was stable for 4 h, whereas both MG and PGG exhibited increased recoveries. In addition, the moderately increased recovery of GA and the dramatically increased recoveries of MG and PGG were observed after incubation at pH 6 for 8 h (Figure 1C). Because MSKE has been reported to be a source of high molecular weight hydrolyzable tannins, such as hexa-, hepta-, octa-, nona-, and deca-*O*-galloylglucose, which are known to be easily degraded into smaller molecules [20], the increased recoveries of GA, MG, and PGG may have been a result of the degradation of other unidentified constituents in MSKE.

### 2.2. Biological Stability in Caco-2 Cell Lysates

**Figure 2:**
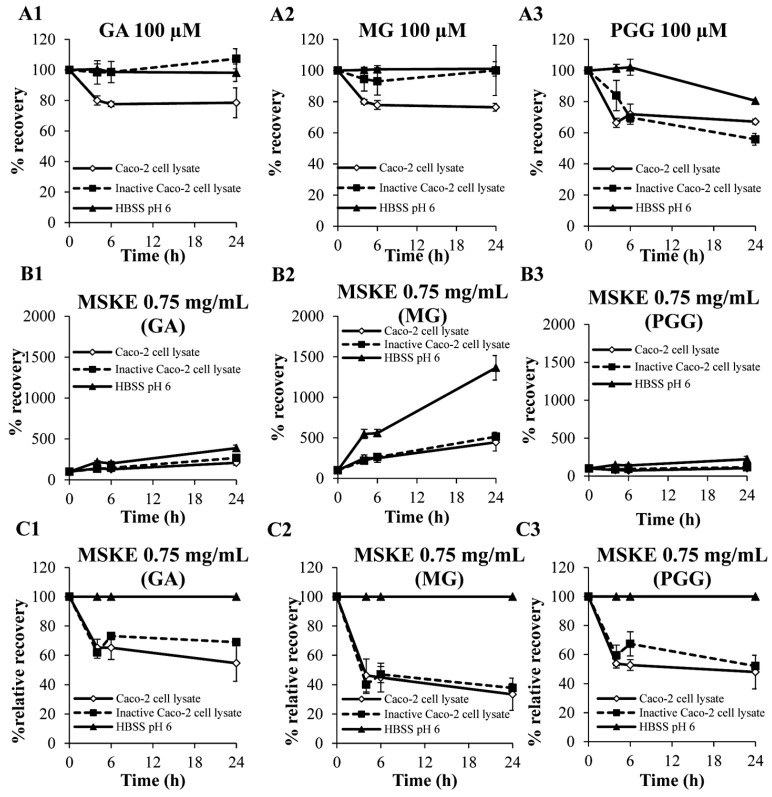
The recoveries of GA, MG and PGG after incubation for 24 h with Caco-2 cell lysates as individual compounds (100 µM for each compounds, **A**) and as principles in 0.75 mg/mL MSKE (**B**). The stabilities of the three phenolic compounds in MSKE are also displayed as the relative recoveries (**C**). Each symbol indicates the mean ± S.D. (*n* = 3).

When incubated individually, the two phenolic compounds were degraded by the cell lysates within 24 h as shown by the reduced recoveries (Figure 2A1,2), whereas both GA and MG were stable after incubation with inactive cell lysates or in HBSS at pH 6 throughout the incubation period. These results implied that the degradation of GA and MG might be caused by the presence of human esterase or conjugation enzymes in the intestinal cells [21]. However, although PGG was stable in HBSS at pH 6 for 6 h and slightly degraded within 24 h, PGG exhibited a decreased recovery after incubation with active and inactive cell lysates, respectively, within 24 h. This result suggested that the decreased recovery of PGG after incubation with the cell lysates and inactive cell lysates could have been caused by the binding of PGG with protein in the lysates [22,23].

Regarding the MSKE principles shown in Figure 2B, the recoveries of the three phenolic compounds increased after incubation for 24 h with HBSS at pH 6 and the two types of lysates. This finding implied that the biological stabilities of GA, MG, and PGG in the cell lysate could have been influenced by the chemical degradation of other unidentified constituents in MSKE at pH 6. However, it could be noticed that the increased recoveries of GA, MG and PGG after incubation with the inactive lysates were lower than those after incubation with HBSS. This result suggested that the unidentified phenolic components in MSKE could be partially stabilized by the interaction with protein or biomolecules in the cell lysates, which masked the reactive moieties on the polyphenol [22,24]. As shown in Figure 2C, although GA, MG, and PGG in MSKE demonstrated decreased relative recoveries after incubation with the cell lysates for 24 h, their relative recoveries were not significantly different from the recoveries after incubation with the inactive lysates, suggesting the inhibition of enzyme activity in the cell lysates. Because plant polyphenols have the ability to inhibit a number of digestive enzymes including β-glucosidase and glucuronosyltransferases [25,26], this result may be a consequent of the enzyme inhibition by other phenolic components in MSKE.

### 2.3. Biological Stability in Rat Fecal Lysates

**Figure 3:**
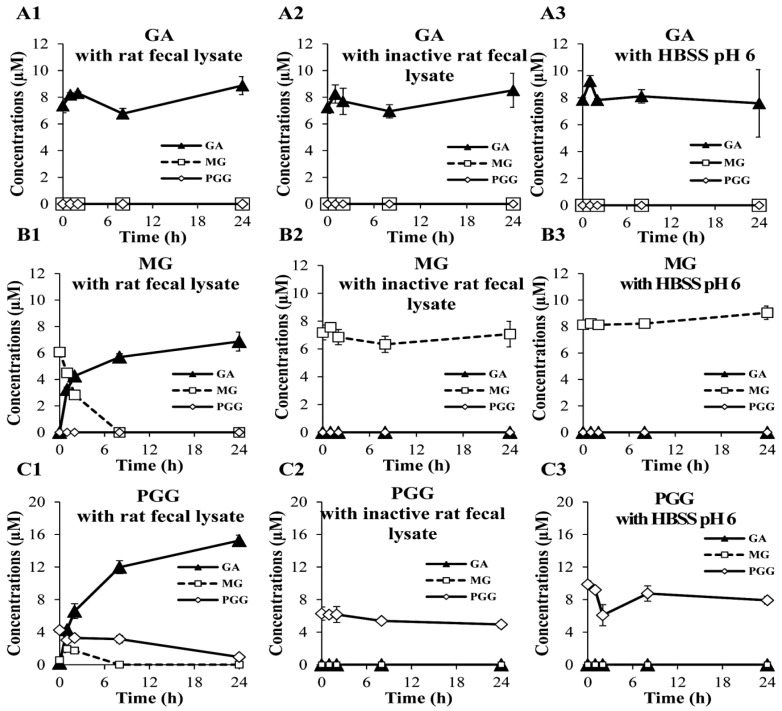
The biological stabilities of GA, MG and PGG at 10 µM for each compound after individual incubation with rat fecal lysates (**A1**, **B1** and **C1**, respectively), inactive rat fecal lysates (**A2**, **B2** and **C2**, respectively) and HBSS at pH 6 (**A3**, **B3** and **C3**, respectively) for 24 h. Each symbol indicates the mean ± S.D. (*n* = 3).

Figure 3 shows the degradations of GA, MG and PGG as individual pure compounds after incubation with rat fecal lysates. When GA was incubated with fecal lysate, inactive lysate, and HBSS at pH 6 for 24 h, GA concentrations were not significantly different from the initial concentrations, and neither MG nor PGG were observed in the same incubation systems. This result suggests that the transformation of GA by rat fecal lysate was not occurred within 24 h. Pyrogallol has been reported to be the gut microbiota metabolite of GA in several studies, however, the biotransformation rate of GA showed high inter-individual differences [27,28,29]. Chen *et al.* [28] demonstrated that the different subjects of human fecal slurries could cause a partial or complete GA degradation observed within 48 h.

On the other hand, the degradation of MG and PGG in the fecal lysates could be observed within 24 h. After incubation with fecal lysates for 24 h, MG continuously decreased until degradation was complete within 8 h, and an increased GA concentration to approximately the initial concentration of MG was observed in the same systems (Figure 3B1). Similarly, PGG exhibited a marked reduction within 24 h in the fecal lysates, and both GA and MG presented increases within the first hour of the same incubation. Additionally, GA exhibited the maximum concentration by approximately 4-fold increase after hydrolysis of PGG. This result was not surprising because five GA moieties are contained in one PGG molecule. After incubation with the inactive lysates or HBSS at pH 6, MG and PGG were stable over 24 h, indicating that the degradation of MG and PGG was caused by enzymes in the fecal lysate, and that GA was the main product of their degradation. This result is consistent with previous studies that MG was hydrolyzed to GA, pyrogallol and resorcinol by rat fecal microflora [30], and PGG was hydrolyzed to GA by tannase, which is a ruminal bacterial enzyme [29]. However, it should be noted that the lower initial concentrations of the three phenolic compounds in the two types of fecal lysates than in HBSS might have been caused by their protein binding properties [22].

**Figure 4:**
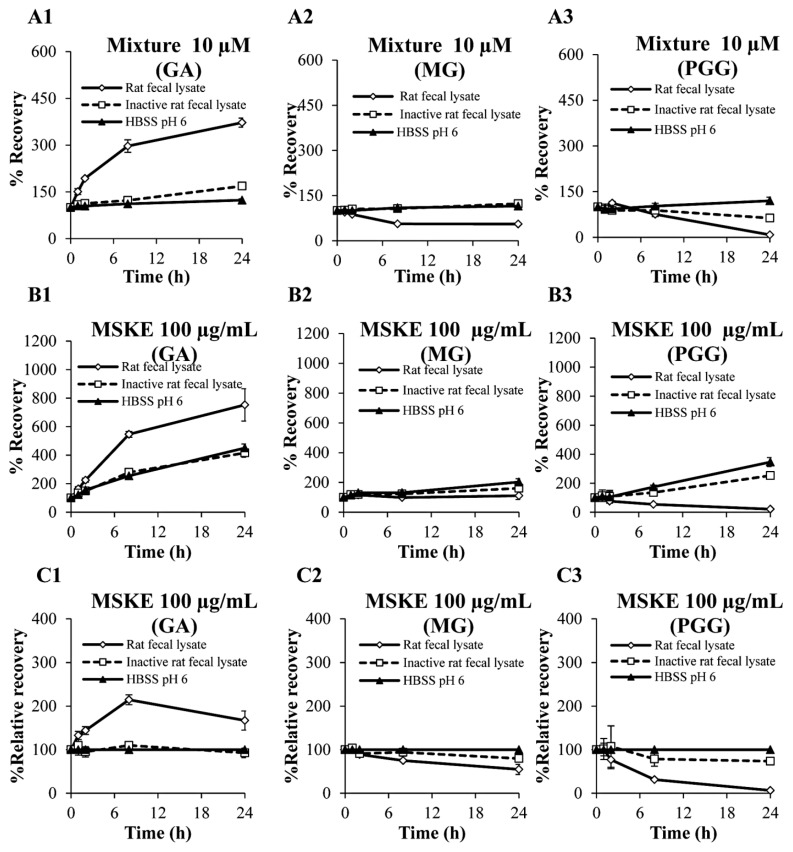
The recoveries of GA, MG and PGG in the mixture (10 µM, **A**) and in MSKE (100 µg/mL, **B**) after incubation with rat fecal lysates. The stabilities of the three phenolic compounds in MSKE are also displayed as the relative recoveries (**C**). Each symbol indicates the mean ± S.D. (*n* = 3).

The degradation of GA, MG and PGG in the mixture after incubation with the fecal lysates yielded results similar to those of the individual compounds after incubation with the same lysates as shown in Figure 4A. After incubation with the fecal lysates for 24 h, the recovery of GA increased to 372.25% ± 14.58%, whereas the recoveries of MG and PGG decreased to 55.41% ± 3.31% and 8.51% ± 1.43%, respectively. When the mixture was incubated with inactive fecal lysates, GA and MG exhibited good stabilities, whereas PGG demonstrated a slightly reduced recovery after 8 h. However, the three phenolic compounds in the mixture incubated with HBSS at pH 6 were stable for 24 h. This finding indicated that PGG was more susceptible to hydrolyze by bacterial enzymes in rat feces than MG.

On the other hand, when MSKE was incubated with fecal lysates for 24 h, the three phenolic compounds showed increased recoveries of GA and MG and decreased recovery of PGG (Figure 4B). In addition, GA, MG and PGG in MSKE displayed increased recoveries after incubation with inactive fecal lysates and HBSS at pH 6, implying that the chemical degradation of other unidentified constituents in MSKE at pH 6 was involved with the biological stabilities of the three phenolic compounds in fecal lysates. Therefore, the biological stabilities of these phenolic compounds were also expressed as the % relative recovery. As shown in Figure 4C, the results revealed similar degradation profiles between the three individual phenolic compounds in MSKE compared with the mixture after incubation with the fecal lysates for 24 h. The relative recovery of GA increased to 167.04% ± 21.95%, whereas the relative recoveries of MG and PGG decreased to 54.67% ± 11.67% and 6.29% ± 1.65%, respectively. In addition, the relative recoveries of GA, MG and PGG in MSKE after incubation with inactive lysates were similar to those after incubation with HBSS, suggested that the other constituents in MSKE could not influence the enzyme activity in the rat feces. However, the hydrolysis of large molecular weight polyphenols, such as ellagitannins, proanthocyanidins and condensed tannins, by bacterial enzymes is the degradation pathway that generates GA as the metabolite [27,28,29,30]. Therefore, the increased GA may result from the degradation of other unidentified polyphenols in MSKE by enzymes in the rat fecal lysate, in addition to the degradation of MG and PGG.

### 2.4. Caco-2 Transepithelial Transport Experiment

**Figure 5:**
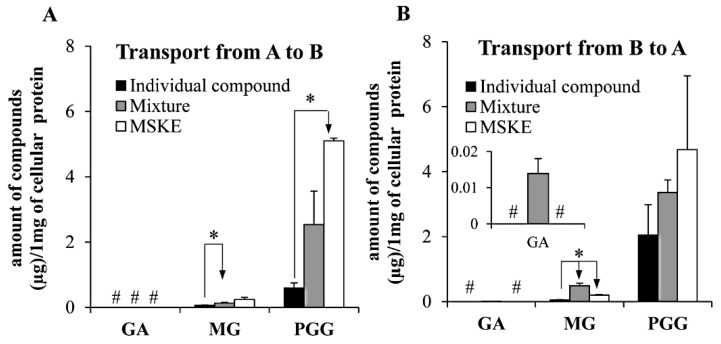
The cellular uptake of GA, MG and PGG after transport as individual compounds, in the mixture and in MSKE across the Caco-2 monolayer from side A to side B (**A**) and from side B to side A (**B**). Each symbol indicates the mean ± S.D. (*n* = 3). ^#^ indicates that the amount of analyte could not be determined. * Significant difference (*p* < 0.05).

The absorption of GA, MG and PGG was investigated individually, in the mixture, and as principles in MSKE using a Caco-2 cell. As individual compounds, MG exhibited the highest apparent permeability coefficient values from the apical (A) to basolateral (B) direction (*P*_app AB_), followed by GA and PGG (Table 1). Similarly, these compounds also showed the same order for the *P*_app BA_ values. Furthermore, the efflux ratios of GA and MG indicate passive absorption mechanism, but the efflux ratio of PGG (> 2) suggests the involvement of an efflux transporter mechanism. These results are consistent with those of Konishi *et al.* [31], who reported that GA permeates through Caco-2 cells via a paracellular route. In addition, Cai *et al.* [32] reported the efflux transport, a multidrug resistance-associated protein 2, appeared to play a role in the transport of PGG with the poor *P*_app AB_ of 0.08 ± 0.03 × 10^−6^ cm/s. As shown in Figure 5, PGG showed the highest accumulation in the cells, followed by MG, whereas the accumulation of GA in the cells could not be detected after individual bi-directional transport across Caco-2 monolayer. Tammela *et al.* [33] demonstrated that polyphenols with strong membrane affinities were generally accompanied by poor absorptive transport, and their permeability and membrane affinity were governed by the degree of hydroxylation, molecular configuration, and alkyl gallate side chain lengths. Therefore, higher cellular accumulation of PGG was attributed to its slow transport and stronger membrane affinity compared with that of MG and the absence of GA accumulated in cells may be due to the paracellular transport mechanism of GA.

When these three compounds were transported in the mixture or MSKE, some of *P*_app AB_ and *P*_app BA_ values presented significant differences (*p* < 0.05) with those of the individual compounds, which resulted in the increased efflux ratios especially for GA and PGG (Table 1). In addition, these three compounds as constituents in the mixture or MSKE showed the increased cellular accumulation trends as compared with the individual transport (Figure 5). These results clearly indicated that some levels of interaction occurred among these phenolic compounds and with the other components in MSKE had an influence on their efflux transport and cellular uptake. Although the mechanisms responsible for the interaction remain unclear, it may have been caused by the inhibition of efflux transporters similar to the increased cellular accumulation of epicatechin after co-incubation with alkyl gallates [34], because alkyl gallates and PGG have been reported to inhibit P-glycoprotein function [35,36]. In addition, the fact that additional GA, MG, PGG and unidentified metabolites during the MSKE transport may occur by the chemical degradation of other constituents in MSKE at pH 6 and by the cellular enzymes could also be the reason for this effect. However, the unknown compounds in MSKE and the metabolites by Caco-2 cell enzymes deserve further investigation.

### 2.5. In Vivo Oral Pharmacokinetic Studies

**Figure 6:**
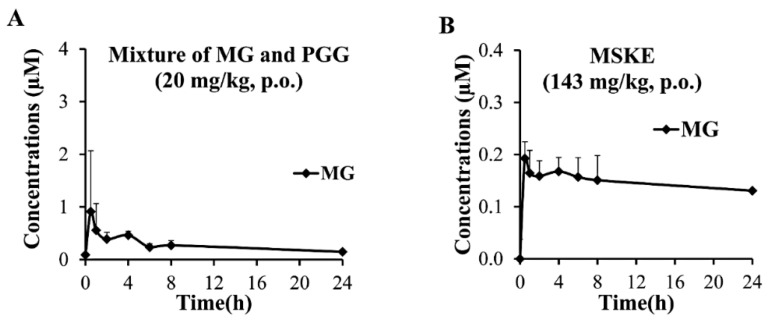
Blood concentration–time curves of MG after oral administration of the mixture with PGG at a dose of 20 mg/kg (**A**) and as principles in MSKE at a dose of 143 mg/kg (**B**). Each symbol indicates the mean ± S.D. (*n* = 5).

The blood concentration–time curves after the oral administration of MG and PGG in the mixture at the dose of 20 mg/kg for each compound is shown in Figure 6A. MG in the mixture induced a double-peak profile, with a second maximum peak at 4 h, which may have been caused by enterohepatic recirculation similar to that of other phenolic compounds, such as genistein [37]. In contrast, blood concentrations of PGG were below the lower limit of quantification (LLOQ, 0.039 µM). Compared with our previous intraperitoneal pharmacokinetic study of this mixture at the same dose [14], blood levels of PGG were also very low, suggesting a first pass metabolism. MG administrated orally exhibited slower absorption (*T*_max_), lower *C*_max_ and lower area under the blood concentration time curves (AUC_0__–24h_) compared with MG administrated intraperitoneally by approximately 3-, 31- and 17-folds, respectively, indicating the route administration effect on the pharmacokinetic parameters of MG. In addition, MG administrated orally also demonstrated a high volume of distribution (*V*_d_) and long *t*_1/2_ compared with MG administrated intraperitoneally by approximately 19- and 1.4-folds, respectively (Table 2), suggesting that MG was distributed widely to the tissues. As shown in Table 3, the accumulation of GA, MG, and PGG could be quantified in the urine and feces between 0 and 24 h after dosing. The total mass recoveries of MG and PGG were lower than 2% after oral administration as the mixture, indicating that >98% of the administered MG and PGG may be metabolized and accumulated in particular organs and tissues after oral administration in rats. MG in the mixture led to higher amounts in the urine relative to that in feces, whereas PGG in the mixture led to lower amounts in the urine compared with those in the feces. This result implied that the parent forms of MG and PGG were primary eliminated from urine and feces of the rats, respectively. In addition, GA found in feces samples confirmed the *in vitro* transformation of MG and PGG by gut microflora, whereas GA found in the urine suggested the digestive and hepatic transformation. 

**Table 1:** The *P*_app_ values of GA, MG and PGG from bi-directional transport across the Caco-2 monolayer.

Analytes	Individual ^a^ (20 μM)			Mixture ^a^ (20 μM)			MSKE ^a^ (0.25 mg/mL)		
	*P*_app_ ± S.D. (×10^−6^ cm/s)		Efflux Ratios	*P*_app_ ± S.D. (×10^−6^ cm/s)		Efflux Ratios	*P*_app_ ± S.D. (×10^−6^ cm/s)		Efflux Ratios
	A to B	B to A		A to B	B to A		A to B	B to A	
GA	1.02 ± 0.36	1.02 ± 0.29	1.00	0.35 ± 0.18 ^b^	1.87 ± 0.47	5.34	1.46 ± 0.21	8.04 ± 0.43 ^b^	5.51
MG	13.57 ± 0.40	7.02 ± 1.11	0.52	12.46 ± 0.63	6.33 ± 0.57	0.51	17.61 ± 1.54 ^b^	11.30 ± 0.31 ^b^	0.66
PGG ^c^	0.28 ± 0.14	0.94 ± 0.14	3.36	0.12 ± 0.09	0.58 ± 0.06 ^b^	4.83	0.83 ± 0.17 ^b^	4.20 ± 0.11 ^b^	5.06

^a^ Data represent the means ± S.D. (*n* = 3). ^b^ Significant differences in the *P*_app_ values of individual compounds (*p* < 0.05). ^c^ *P*_app_ values of PGG were determined between 180 and 240 min.

**Table 2:** Pharmacokinetic parameters of MG and PGG after oral administration of the mixture of MG and PGG or the administration of MSKE in rats (*n =* 5).

Pharmacokinetic Parameters	Mixture of MG and PGG (20 mg/kg)			MSKE (143 mg/kg; 20 mg/kg PGG and 0.13 mg/kg MG)	
	i.p. (*n* = 5) ^b^	p.o. ^a^		p.o. ^a^	
	MG	MG	PGG	MG	PGG
*T*_max_ (h)	0.85 ± 0.70	2.30 ± 1.64	ND	0.50 ± 0.00	ND
*C*_max_ (µM)	34.72 ± 17.32	1.13 ± 1.06	ND	0.24 ± 0.06	ND
AUC_0–24h_ (h·µM)	109.9 0 ± 73.40	6.47 ± 0.75	ND	3.57 ± 0.79	ND
*K*_e_ (1/h)	0.056 ± 0.032	0.036 ± 0.016	ND	0.015 ± 0.008	ND
*t*_1/2_ (h)	17.50 ± 12.25	24.84 ± 15.71	ND	65.17 ± 49.35	ND
*V*_d_ (L/kg)	530.95 ± 247.54	10,246.44 ± 2660.70	ND	135.39 ± 21.94	ND
CL (L/h/kg)	159.91 ± 76.05	332.06 ± 99.72	ND	2.11 ± 1.26	ND
MRT_last_ (h)	8.71 ± 2.53	8.69 ± 1.57	ND	11.06 ± 0.60	ND

^a^ Data represent the means ± S.D. (*n* = 5).^b^ Pharmacokinetic parameters of MG after intraperitoneal injection (i.p.) were reported by Jiamboonsri *et al.* [14]. ND = Parameter could not be determined. *K*_e_, Elimination rate constant; CL, Total blood clearance; MRT_last_, Mean residence time of the unchanged drug in the systemic circulation.

**Figure 7:**
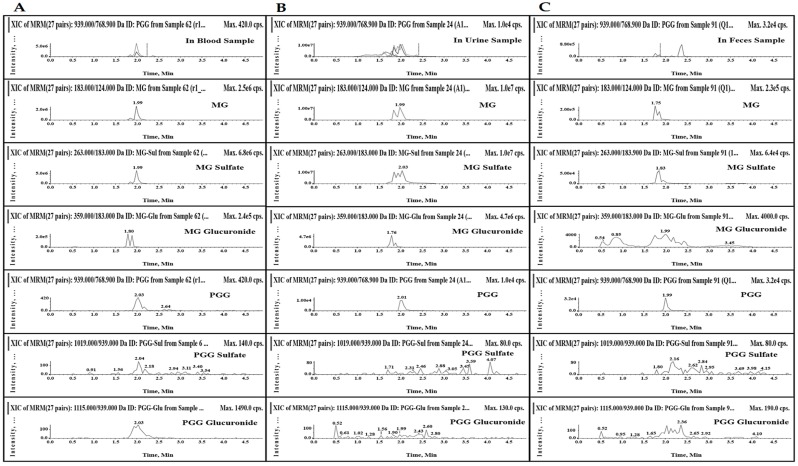
The representative MRM chromatograms of MG, PGG and their sulfate and glucuronide metabolites in blood sample collected at 30 min (**A**) and urine samples (**B**) and fecal samples (**C**) between 0 and 24 h after oral administration of the mixture of MG and PGG at a dose of 20 mg/kg for each compound.

Krook and Hagerman [24] demonstrated that GA, pyrogallol and galloyl esters of glucose were the decomposition products of PGG detected *in vitro* digestion model mimicking the human digestive system. Similarly, GA was also the main metabolite of MG in a rat liver perfusion study [38]. Moreover, MG has been reported to be glucuronated by rat and human liver microsomes [39,40]. Therefore, the samples collected after oral administration of the mixture were selected to preliminarily investigate some specified metabolites of MG and PGG. Figure 7 shows the representative multiple reaction monitoring (MRM) chromatograms of MG, PGG and their metabolites in blood at 30 min, in urine (0−24 h) and in feces (0−24 h) samples. The chromatograms reveal MG sulfates in the blood, urine and feces samples, whereas MG glucuronides were observed in the blood and urine samples. However, PGG sulfate was not observed in the samples, whereas PGG glucuronide was only observed in the blood sample at the low intensity. Therefore, this results suggested that phase II conjugation may be involved the metabolism of MG and PGG *in vivo*. However, although the *in vivo* pharmacokinetic of GA and its metabolites were not elucidated in this study due to limitation of the ultra-performance liquid chromatography-tandem mass spectrometric (UPLC–MS/MS) method, MG and PGG could possibly generate other metabolites similar to GA, such as resorcinol, pyrogallol, 4-*O*-methylgallic acid, pyrogallol-1-*O*-β-d-glucuronide, 4-*O*-methylgallic acid-3-*O*-sulfate and 2-*O*-methylpyrogallol [9,10,11,12,13,41]. 

When MSKE at a dose of 143 mg/kg (0.13 and 20 mg/kg of MG and PGG, respectively) was administrated in rats, MG exhibited pharmacokinetic parameters in a dose−dependent manner (Figure 6B and Table 2), whereas blood levels of PGG remained below the limit of quantification. Similar to the excretion results of the mixture, the amount of PGG in MSKE found in the urine was not significantly different (*p* > 0.05) from that in the mixture recovered from the urine (Table 3). This result suggests that the other components in the extract could not intensify the blood levels or the urinary excretion of PGG. However, it could be noted that the amounts of GA, MG, and PGG in MSKE found in the feces were significantly higher (*p* < 0.05) than those in the mixture recover in the feces. This result may be explained by the metabolism of MG, PGG and other unidentified components in MSKE by microflora similar to the *in vitro* results of MSKE in the fecal lysate. However, although the *in vitro* results of chemical degradation, biological degradation, and absorption could be partially explained the reasons underlying the low oral bioavailability of GA, MG, and PGG, and also demonstrated the effects of co-components in MSKE on their pharmacokinetic behaviors, further investigation of their metabolism, tissue distribution and multiple dose administration are necessary to understand their pharmacokinetic characteristics *in vivo*.

**Table 3:** Excretion of the three analytes in urine and feces after oral administration of the mixture of MG and PGG or the administration of MSKE in rats (*n =* 5).

Total Mass (µg)	Analytes	Oral Administration ^a^	
		Mixture of MG and PGG (20 mg/kg)	MSKE (143 mg/kg; 20 mg/kg PGG and 0.13 mg/kg MG)
Urine (0−24 h)	GA	42.95 ± 26.88	54.54 ± 26.59
	MG	100.36 ± 74.67	9.81 ± 2.81 ^b^
	PGG	3.73 ± 1.41	3.02 ± 0.92
Feces (0−24 h)	GA	9.64 ± 1.91	39.47 ± 17.44 ^b^
	MG	3.25 ± 1.73	18.35 ± 10.14 ^b^
	PGG	28.20 ± 10.14	85.66 ± 25.53 ^b^
% recovery	MG	1.89 ± 1.39	73.38 ± 29.98 ^b^
	PGG	0.56 ± 0.15	2.61 ± 2.66

^a^ Data represent the means ± S.D. (*n* = 5). ^b^ Significant difference (*p* < 0.05) in the total mass of the same analyte after oral administration of the mixture.

## 3. Experimental Section

### 3.1. Materials

Gallic acid (GA; ≥98% purity) and methyl gallate (MG; ≥98% purity) were purchased from Fluka (Buchs, Switzerland). Pentagalloylglucopyranose (PGG; >95% purity) was purchased from Endotherm GmbH (Saarbrücken, Germany). Formononetin, which was used as an internal standard (I.S.) for LC–MS/MS, was purchased from LC Laboratories (Woburn, MA, USA). HBSS (powder form) was purchased from Sigma-Aldrich (St. Louis, MO, USA). The Ora-Plus oral suspending vehicle was purchased from Perrigo (Dublin, Ireland). All solvents used in the UPLC−MS/MS were of LC−MS grade and obtained from EMD Millipore (Gibbstown, NJ, USA). All other materials (typically analytical grade or higher) were used as received.

### 3.2. Preparation of MSKE

Fully grown, unripened Thai mango fruits (*Mangifera indica* L. cv. “Fahlun”) were purchased from a local market. A voucher specimen (R.B. 20007) was deposited in the Museum of Natural Medicine, Faculty of Pharmaceutical Sciences, Chulalongkorn University, Bangkok, Thailand. Kernels were extracted according to the method described by Nithitanakool *et al.* [4]. Briefly, the chopped kernels were homogenized in hot ethanol (80 °C) and defatted with hexane. After the solvents were evaporated, the remaining aqueous portion was freeze-dried and stored in an airtight container at −20 °C until use. For further experiments in this study, the concentration of MSKE was used at the equivalent concentration of PGG because PGG was contained a relatively high amount in MSKE and had the highest pharmacological activities [4,5,6,7,8].

### 3.3. Standardization of Plant Extract

**Figure 8:**
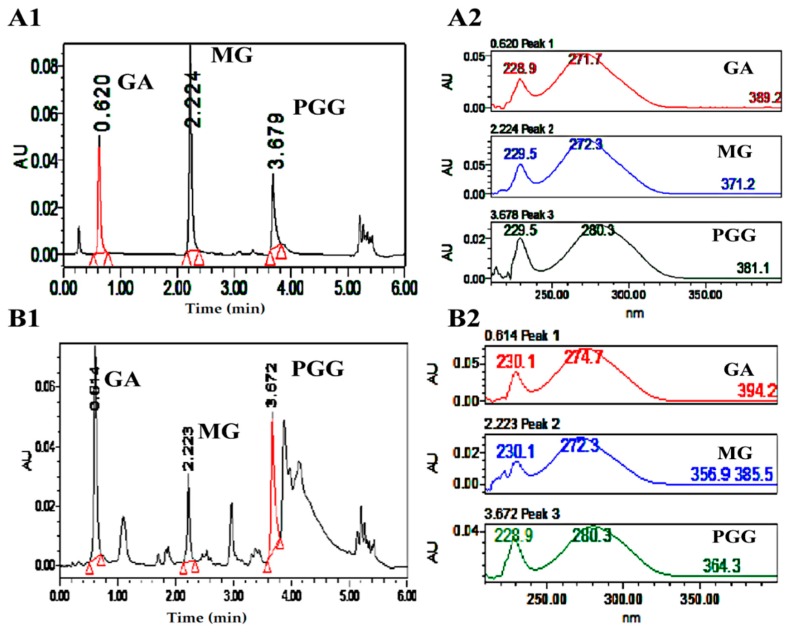
UPLC chromatograms, and UV spectra of GA, MG, and PGG as the reference standards (**A1** and **A2**) and as principles in MSKE (**B1** and **B2**).

UPLC was used to quantify the contents of GA, MG, and PGG in MSKE; compounds were identified based on a comparison of the retention times and UV spectra of the unknown sample peaks with that of the reference standards. The retention times were 0.6 min for GA, 2.2 min for MG, and 3.6 min for PGG (Figure 8). The UPLC method validations were performed according to the International Conference on Harmonization (ICH) guidelines. The linear regression equations for GA and MG in the concentration range of 0.15–40 µM with an r^2^ of 0.9998 were Y = 7450.8X−1108 and Y = 8620.4X−2385, respectively. For PGG, the linear regression equation in the concentration range of 1.25–40 µM was Y= 5730.8X−883.94 with an r^2^ of 0.9993. The limit of detection (LOD) and limit of quantitation (LOQ) for GA and MG were 0.08 and 0.15 µM, respectively, and the LOD and LOQ for PGG were 0.63 and 1.25 µM, respectively. The precision and accuracy were determined at 0.2, 2, and 20 µM for GA and MG and at 0.4, 4, and 40 µM for PGG. The accuracy, which was expressed as the percent recovery of an analytical method, for GA, MG, and PGG was in the range of 91.45% ± 0.53% to 118.30% ± 0.73%, whereas the intra-day and inter-day precision, which was expressed as the percentage of coefficient of variation (% CV), was in the range of 0.34%–1.68%. The amounts of GA, MG, and PGG in MSKE were 1.53% ± 0.04%, 0.09% ± 0.01%, and 14.03%± 0.77% *w*/*w* based on dry weights, respectively.

### 3.4. Cell Culture

Caco-2 TC7 cells were kindly provided by Prof. Monique Rousset of INSERM U178 (Villejuif, France). The Caco-2 cells used in this study were between passages 42−48 and were cultured according to the method described by Hu *et al.* [42].

### 3.5. Animals

Male SD rats (260–330 g, 8–12 weeks old) were acquired from Harlan Laboratories, Inc. (Indianapolis, IN, USA). All animals were maintained in an environmentally controlled room (25 ± 2 °C, 50% ± 5% humidity, and 12 h dark-light cycle) for at least 1 week before performing any experiments. All procedures involving laboratory animal use were conducted in accordance with the internationally accepted principles for laboratory animal use and care (the US guidelines) and approved by the University of Houston’s Institutional Animal Care and Use Committee (No. 12-031).

### 3.6. Chemical and Biological Stabilities

#### 3.6.1. Chemical Stability in HBSS pH 5, 6, 7 and 8

The chemical stability of GA, MG, PGG and MSKE was determined in HBSS at pH 5, 6, 7 and 8. The test compounds were added to HBSS to obtain the final concentrations (2 and 20 μM) for each standard compound or MSKE (0.25 mg/mL), which is equivalent to concentrations of 26.70 ± 1.35 µM GA, 4.90 ± 0.38 µM MG and 27.09 ± 8.01 µM PGG. The solutions at pH 5, 7 and 8 were then incubated in a water bath at 37 °C for 4 h, whereas the solutions at pH 6 were incubated for 8 h. Samples (200 μL) were collected at different time intervals (0, 1, 2, 3 and 4 h or 0, 1, 2, 4, 6 and 8 h) and then vortexed and centrifuged at 15,500 rpm for 15 min. The supernatant was injected into the UPLC for analysis. All experiments were performed in triplicate.

#### 3.6.2. Biological Stability in Caco-2 Cell Lysates

Caco-2 cells were cultured in T75 flasks at a seeding density of 2 × 10^6^ cells/flask for 12–15 days. The cell lysate was freshly prepared by washing the cells twice with ice-cold HBSS at pH 6 (12 mL). The cells were then collected by scraping and resuspending in ice-cold HBSS at pH 6 (7.5 mL/flask). The cell suspension was vortexed and sonicated in an ice water bath for 30 min to obtain the lysate, which was kept on ice until use, and its cellular protein concentration was determined using a BCA™ protein assay kit (Thermo Fisher Scientific, Rockford, IL, USA) with bovine serum albumin as a reference standard.

Many phenolic compounds were subject to sulfation and glucuronidation by Caco-2 cells at concentrations higher than 20 μM [16,21,43] and the metabolites of GA, MG and PGG could not be observed in Caco-2 transport experiment. Therefore, the concentration of the standard pure compounds used in this study was higher than that used in the chemical stability experiment. After preparing the cell lysate, the test compounds were prepared by dissolution in HBSS at pH 6 and then added to the lysates for a final concentration of 1.08 mg/mL protein to obtain final individual concentrations of 100 μM for GA, MG and PGG and 0.75 mg/mL for MSKE, which is equivalent to concentrations of 55.12 ± 4.57 µM GA, 7.56 ± 0.85 µM MG and 110.02 ± 17.52 µM PGG. The mixtures were then incubated in a water bath at 37 °C for 24 h, and samples (50 µL for standard compounds, and 200 µL for MSKE) were collected at 0, 4, 6 and 24 h. Each sample was then added to an ice-cold 50% methanol (150 µL for GA, MG and PGG, and 600 µL for MSKE) to stop the reaction, vortexed and centrifuged at 15,500 rpm for 15 min, and the supernatant was injected into the UPLC for analysis. The experiments were performed in triplicate, and for each test, HBSS at pH 6 was used as a blank control, and the inactive cell lysate was prepared by heating at 95 °C for 15 min and then used as a negative control.

#### 3.6.3. Biological Stability in Rat Fecal Lysates

The rat fecal lysate was prepared according to the method of Niu *et al.* [44] with slight modifications. Briefly, 3 g of feces was collected from SD rats (8–10 weeks, *n* = 6) were then washed with ice-cold 0.1 mM potassium phosphate buffer (KPi, 10 mL). After resuspension in ice-cold 0.1 mM KPi (20 mL), the feces were vortexed and sonicated in an ice water bath for 45 min. To obtain the fecal lysate, the mixture was centrifuged at 15,000 rpm at 4 °C for 30 min. The supernatant was collected and maintained at –80 °C until use. The protein concentration in the fecal lysate was determined using a BCA™ protein assay kit.

The prepared fecal lysate was thawed and diluted with ice-cold HBSS at pH 6 to obtain a final concentration of 0.5 mg/mL protein for each test. Because the hydrolysis of MG and PGG by fecal lysates was expected to generate the higher concentration of GA, the concentration of standard pure compounds used in this study was lower than that used in the chemical stability in order to analysis all compounds in the same time without sample dilution. The test solutions prepared in HBSS at pH 6 were added to the lysates at the following final concentrations: 10 μM of GA, MG and PGG as individual compounds, 10 μM of each compound in the mixture of GA, MG and PGG, and 100 μg/mL of MSKE, which is equivalent to concentrations of 7.08 ± 0.43 μM GA, 3.54 ± 0.24 μM MG and 11.04 ± 0.66 μM PGG. The mixtures were then incubated in water bath at 37 °C for 24 h. After samples (200 μL) were collected at each specific time point (0, 1, 2, 8, and 24 h), the reaction was stopped by adding ice-cold methanol (50 μL). The samples were then vortexed and centrifuged at 15,500 rpm for 15 min, and the supernatant was injected into the UPLC for analysis. The experiments were performed in triplicate, and in each test, inactive fecal lysates, which were prepared by heating at 95 °C for 15 min, and HBSS at pH 6 were used as a negative control and blank control, respectively.

#### 3.6.4. Data Analysis in Chemical and Biological Stability Assays

The chemical and biological stabilities of the phenolic compounds were expressed as the concentration of the analyte at different time points relative to the concentration of the same analyte at time zero multiplied by 100 (% recovery). The biological stabilities of the phenolic compounds in MSKE were also assessed by comparing the recoveries of the analytes after incubation with either the lysate or inactive lysate to the recovery after incubation with HBSS at each time point multiplied by 100 (% relative recovery).

### 3.7. Caco-2 Transepithelial Transport Experiment

The transport experiment using Caco-2 monolayer was performed according to the criteria in our laboratory as previously reported with slight modification [45]. Briefly, the cell monolayers were prepared by seeding 400,000 cells onto a polycarbonate insert, which has a surface area and pore size of 4.2 cm^2^ and 3 μm, respectively (Nunc™, Thermo Fisher Scientific, Waltham, MA, USA). Cells were maintained in a humidified atmosphere at 37 °C (90% humidity and 5% CO_2_) and fed every other day. After 19 to 22 days post-seeding, the cell monolayers were used in the experiments. Before the experiment, the monolayers were quickly washed twice with pre-warmed HBSS at pH 7.4 (37 °C). The integrity of each monolayer was then verified by measuring the transepithelial electrical resistance (TEER) using a Millicell^®^ ERS meter (Millipore, Bedford, MA, USA), and layers with TEER values less than 400 Ω/cm^2^ relative to that of the blank insert were discarded. Then, the monolayers were incubated with the HBSS buffer at 37 °C for 1 h, and the incubation buffer was then aspirated. The test compounds were dissolved in HBSS at pH 6 to obtain final concentrations for the individual compounds (20 μM), mixture (20 μM) and MSKE (0.25 mg/mL), which were then loaded onto the A or B side (2.6 mL) as the donor side. A 2.6 mL aliquot of HBSS at pH 6 without the test compounds was loaded into the other side as the receiver side. After incubation at 37 °C, 0.6 mL of sample was collected from the receiver side at indicated times (60, 120, 180 and 240 min), and the same volume of receiver medium was replaced after each sampling. At the end of the experiment, the cells were washed twice with ice-cold HBSS at pH 6. The cells in each well were then collected and resuspended in 600 μL of HBSS at pH 6, and sonicated in an ice water bath at an output of 9 W for 30 min to obtain the cell lysates. The lysate was divided into the following aliquots: (1) 20 μL for quantitative protein assessment using a BCA™ protein assay kit and (2) 580 μL for quantitative test compounds. The samples collected from the transport study were stored at −20 °C until the UPLC or LC–MS/MS analysis. The experiments were performed in triplicate.

#### Data Analysis in the Caco-2 Cell Culture Model

The apparent permeability coefficient (*P*_app_, cm/s) of each compound was calculated according to the following equation (Equation (1)):

(1) $$P_{\text{app}}=\;\frac{\text{dQ}}{\text{dt}}\times \frac{1}{{\text{AC}}_{0}}$$

where dQ/dt is the compound permeation rate (nmol/s), A denotes the membrane surface area, and C_0_ is the initial concentration on the donor side (µM). The efflux ratio was calculated according to the following equation (Equation (2)):

(2) $$\text{Efflux ratio}=\;\frac{P_{\text{app BA}\left(\text{mean}\right)}}{P_{\text{app AB}\left(\text{mean}\right)}}$$

where *P*_app BA_ (mean) and *P*_app AB_ (mean) are the average apparent permeability coefficients of each compound from side B to side A, and from side A to side B, respectively.

### 3.8. In Vivo Oral Pharmacokinetic Studies

SD rats (five rats/group) were individually housed in stainless-steel metabolic cages, which allowed for the separation and collection of urine and feces. The rats were fasted overnight with *ad libitum* access to water prior to the day of the experiment. After a rat was anesthetized with isoflurane gas, the prepared suspension of test compound in an oral suspension vehicle was administered to the animals via oral gavage at a dose of 20 mg/kg for each compound in the mixture of MG and PGG or at 143 mg/kg MSKE, which is equivalent to approximate doses of 20 mg/kg PGG and 0.13 mg/kg MG. Subsequently, the rats’ tails were snipped near the tip to collect blood samples (approximately 50 µL), which were collected in heparinized tubes at 0, 0.50, 1, 2, 4, 6, 8 and 24 h. To investigate the excretion of phenolic compounds, urine and feces were collected between 0–24 h post-administration into tubes containing penicillin G (200 mg) and dry tubes, respectively, which were pre-weighted before use. After completing the pharmacokinetic study, the urine volume and dry feces weights were recorded, and all samples were stored at −80 °C until analysis.

#### 3.8.1. Sample Preparation Procedure

The blood and urine samples were prepared according to our previously reported methods [14]. The fecal samples were prepared by adding 30 mL methanol to the sample, and the mixture was then vortexed and sonicated for 30 min to obtain the fecal lysates, which were extracted via the same method.

#### 3.8.2. Pharmacokinetic Analysis

The pharmacokinetic parameters of MG and PGG were calculated based on the non-compartmental method using WinNonlin 3.3 (Pharsight Corporation, Mountain View, CA, USA). The total mass of analytes was calculated according to the following equations (Equations (3)–(6)):

The mass recovery (%) = (total mass in blood + total mass in urine + total mass in feces)/initial mass of analyte
(3)

Total mass in blood = C_24h_ in blood × rat blood volume (70 mL/kg of body weight) (4)

Total mass in urine = C_0–24h_ in urine × volume of urine (0–24 h) (5)

Total mass in feces = C_0–24h_ in fecal lysate × volume of fecal lysate (30 mL) (6)

where C_24h_ in the blood is the concentration of analyte found in the blood sample at 24 h, and C_0–24h_ in the urine and C_0–24h_ in the fecal lysate are the concentrations of the analyte found in the urine and fecal samples, respectively, between 0 and 24 h.

### 3.9. UPLC Analysis of GA, MG and PGG

A UPLC was performed using an Acquity^TM^ system (Waters, Milford, MA, USA) equipped with a diode-arrayed detector and Empower software. The UPLC conditions for analyzing GA, MG, and PGG in the aqueous samples were Kinetex C18 column at 50 mm × 2.1 mm I.D. and 5 µm particle size (Phenomenex Inc., Torrance, CA, USA); mobile phase A at 0.5% formic acid in deionized water; mobile phase B at 100% acetonitrile; gradient at 0.0–1.0 min (0% B), 1.0–4.8 min (0%–30% B), 4.8–5.0 min (30%–95% B), 5.0–5.5 min (95%–0% B), and 5.5–6.0 min (0% B); flow rate at 0.55 mL/min; column temperature at 40 °C; sample temperature at 20 °C; and injection volume at 10 µL. UV detection was performed by monitoring the absorbance signal at 280 nm.

### 3.10. LC–MS/MS Analysis of MG and PGG

A Waters UPLC system coupled with an ESI triple quadrupole mass spectrometer (QTRAP5500, AB Sciex, Foster City, CA, USA) was used to analyze MG and PGG in biological samples. The LC–MS/MS conditions were previously published [14]. The UPLC conditions were Kinetex C18 column at 50 mm × 2.1 mm I.D. and 5 µm particle size (Phenomenex Inc.); mobile phase A at 0.1% formic acid in deionized water; mobile phase B at 100% acetonitrile; gradient at 0.0–0.5 min (0% B), 0.5–1.0 min (0%–30% B), 1.0–2.0 min (30%–90% B), 2.0–3.0 min (90% B), 3.0–3.5 min (90%–0% B) and 3.5–5.0 min (0% B); flow rate at 0.5 mL/min; column temperature at 40 °C; sample temperature at 20 °C; and injection volume at 10 µL. The mass spectrometer employed an MRM method in the negative ion mode. The instrument-dependent parameters for obtaining the mass spectra were set as follows: ionspray voltage at −4.5 kV; ion source temperature at 550 °C; gas 1 at 60 psi; gas 2 at 60 psi; and curtain gas at 20 psi. The unit mass resolution was set in both mass-resolving quadruples Q1 and Q3. Because of the absence of authentic standard for the glucuronide and sulfate metabolites of MG and PGG, qualification of the metabolites in the biological samples was accomplished using the compound-dependent parameters for MG and PGG. The compound-dependent parameters are summarized in Table 4.

### 3.11. Statistical Analysis

All of the experimental results are expressed as the mean ± standard deviation (S.D.). All of the statistical analyses were performed using SPSS (version 17.0, SPSS Inc., Chicago, IL, USA). An analysis of variance (ANOVA) was performed, and significant differences between means were determined using Tukey’s honesty significant difference test or Dunnett’s T3 test at a significance level of *p* < 0.05.

**Table 4:** Compound-dependent parameters in the UPLC−MS analysis.

Analytes	Q1 (*m/z*)	Q3 (*m/z*)	DP (V)	CE (V)	CXP (V)	Dwell Time (ms)
MG	183	124	−98	−30	−9	100
MG sulfate	263	183	−98	−30	−9	100
MG glucuronide	359	183	−98	−30	−9	100
PGG	939	769	−194	−44	−39	100
PGG sulfate	1019	939	−194	−44	−39	100
PGG glucuronide	1115	939	−194	−44	−39	100
Formononetin	267	252	−54	−28	−11	100

## 4. Conclusions

This study demonstrates that chemical degradation under high pH conditions, biological degradation by intestinal cell or gut microflora enzymes and poor absorptive permeability are the potential factors affecting the bioavailability of the key phenolic principles in MSKE, especially the low oral bioavailability of PGG, which is a major phenolic compound in MSKE. In addition, the results clearly showed that the other unidentified constituents of MSKE could not promote the blood levels of PGG after a single dose oral administration; however, they might influence the elimination of PGG and the pharmacokinetic characteristics of the other key phenolic compounds in MSKE. This pharmacokinetic study is also valuable for illustrating the potential development of MSKE and its phenolic principles as natural therapeutic agents.

## References

[1] Ribeiro S.M.R., Barbosa L.C.A., Queiroz J.H., Knödler M., Schieber A. (2008). Phenolic compounds and antioxidant capacity of Brazilian mango (*Mangifera indica* L.) varieties. Food Chem..

[2] Abdalla A.E.M., Darwish S.M., Ayad E.H.E., El-Hamahmy R.M. (2007). Egyptian mango by-product 1. Compositional quality of mango seed kernel. Food Chem..

[3] Berardini N., Carle R., Schieber A. (2004). Characterization of gallotannins and benzophenone derivatives from mango (*Mangifera indica* L. cv. “Tommy Atkins”) peels, pulp and kernels by high-performance liquid chromatography/electrospray ionization mass spectrometry. Rapid Commun. Mass Spectrom..

[4] Nithitanakool S., Pithayanukul P., Bavovada R. (2009). Antioxidant and hepatoprotective activities of Thai mango seed kernel extract. Planta Med..

[5] Jiamboonsri P., Pithayanukul P., Bavovada R., Chomnawang M.T. (2011). The inhibitory potential of Thai mango seed kernel extract against methicillin-resistant *Staphylococcus aureus*. Molecules.

[6] Nithitanakool S., Pithayanukul P., Bavovada R., Saparpakorn P. (2009). Molecular docking studies and anti-tyrosinase activity of Thai mango seed kernel extract. Molecules.

[7] Leanpolchareanchai J., Pithayanukul P., Bavovada R., Saparpakorn P. (2009). Molecular docking studies and anti-enzymatic activities of Thai mango seed kernel extract against snake venoms. Molecules.

[8] Pithayanukul P., Leanpolchareanchai J., Saparpakorn P. (2009). Molecular docking studies and anti-snake venom metalloproteinase activity of Thai mango seed kernel extract. Molecules.

[9] Konishi Y., Hitomi Y., Yoshioka E. (2004). Intestinal absorption of *p*-coumaric and gallic acids in rats after oral administration. J. Agric. Food Chem..

[10] Ferruzzi M.G., Lobo J.K., Janle E.M., Cooper B., Simon J.E., Wu Q.L., Welch C., Ho L., Weaver C., Pasinetti G.M. (2009). Bioavailability of gallic acid and catechins from grape seed polyphenol extract is improved by repeated dosing in rats: Implications for treatment in Alzheimer’s disease. J. Alzheimers Dis..

[11] Song R., Xu L., Zhang Z., Tian Y., Xu F., Dong H. (2010). Determination of gallic acid in rat plasma by LC-MS-MS. Chromatographia.

[12] Shahrzad S., Bitsch I. (1998). Determination of gallic acid and its metabolites in human plasma and urine by high-performance liquid chromatography. J. Chromatogr. B Biomed. Sci. Appl..

[13] Shahrzad S., Aoyagi K., Winter A., Koyama A., Bitsch I. (2001). Pharmacokinetics of gallic acid and its relative bioavailability from tea in healthy humans. J. Nutr..

[14] Jiamboonsri P., Pithayanukul P., Bavovada R., Gao S., Hu M. (2015). A validated liquid chromatography-tandem mass spectrometry method for the determination of methyl gallate and pentagalloyl glucopyranose: Application to pharmacokinetic studies. J. Chromatogr. B Anal. Technol. Biomed. Life Sci..

[15] Li L., Shaik A.A., Zhang J., Nhkata K., Wang L., Zhang Y., Xing C., Kim S.H., Lü J. (2011). Preparation of penta-*O*-galloyl-β-d-glucose from tannic acid and plasma pharmacokinetic analyses by liquid–liquid extraction and reverse-phase HPLC. J. Pharm. Biomed. Anal..

[16] Gao S., Hu M. (2010). Bioavailability challenges associated with development of anti-cancer phenolics. Mini Rev. Med. Chem..

[17] Chang Q., Zuo Z., Ho W.K., Chow M.S. (2005). Comparison of the pharmacokinetics of hawthorn phenolics in extract versus individual pure compound. J. Clin. Pharmacol..

[18] Chen L., Lee M.J., Li H., Yang C.S. (1997). Absorption, distribution, elimination of tea polyphenols in rats. Drug Metab. Dispos..

[19] Friedman M., Jürgens H.S. (2000). Effect of pH on the stability of plant phenolic compounds. J. Agric. Food Chem..

[20] Engels C., Gänzle M.G., Schieber A. (2012). Fast LC–MS analysis of gallotannins from mango (*Mangifera indica* L.) kernels and effects of methanolysis on their antibacterial activity and iron binding capacity. Food Res. Int..

[21] Kern S.M., Bennett R.N., Needs P.W., Mellon F.A., Kroon P.A., Garcia-Conesa M.T. (2003). Characterization of metabolites of hydroxycinnamates in the *in vitro* model of human small intestinal epithelium Caco-2 cells. J. Agric. Food Chem..

[22] Jakobek L. (2015). Interactions of polyphenols with carbohydrates, lipids and proteins. Food Chem..

[23] Hagerman A.E., Rice M.E., Ritchard N.T. (1998). Mechanisms of protein precipitation for two tannins, pentagalloyl glucose and epicatechin_16_ (4→8) catechin (procyanidin). J. Agric. Food Chem..

[24] Krook M.A., Hagerman A.E. (2012). Stability of polyphenols epigallocatechin gallate and pentagalloyl glucose in a simulated digestive system. Food Res. Int..

[25] Griffiths D.W. (1986). The inhibition of digestive enzymes by polyphenolic compounds. Adv. Exp. Med. Biol..

[26] Jenkinson C., Petroczi A., Naughton D.P. (2013). Effects of dietary components on testosterone metabolism via UDP-glucuronosyltransferase. Front. Endocrinol..

[27] Mena P., Calani L., Bruni R., Rio D.D., Tuohy K., Rio D.D. (2015). Bioactivation of high-molecular-weight polyphenols by the gut microbiome. Diet-Microbe Interactions in the Gut: Effects on Human Health and Disease.

[28] Chen H., Hayek S., Rivera Guzman J., Gillitt N.D., Ibrahim S.A., Jobin C., Sang S. (2012). The microbiota is essential for the generation of black tea theaflavins-derived metabolites. PLoS ONE.

[29] Bhat T.K., Singh B., Sharma O.P. (1998). Microbial degradation of tannins–A current perspective. Biodegradation.

[30] Brown J.P., Rowland I.R. (1988). Hydrolysis of glycosides and esters. Role of the Gut Flora in Toxicity and Cancer.

[31] Konishi Y., Kobayashi S., Shimizu M. (2003). Transepithelial transport of *p*-coumaric acid and gallic acid in Caco-2 cell monolayers. Biosci. Biotech. Biochem..

[32] Cai K., Hagerman A.E., Minto R.E., Bennick A. (2006). Decreased polyphenol transport across cultured intestinal cells by a salivary proline-rich protein. Biochem. Pharmacol..

[33] Tammela P., Laitinen L., Galkin A., Wennberg T., Heczko R., Vuorela H., Slotte J.P., Vuorela P. (2004). Permeability characteristics and membrane affinity of flavonoids and alkyl gallates in Caco-2 cells and in phospholipid vesicles. Arch. Biochem. Biophys..

[34] Tagashira T., Choshi T., Hibino S., Kamishikiryou J., Sugihara N. (2012). Influence of gallate and pyrogallol moieties on the intestinal absorption of (−)-epicatechin and (−)-epicatechin gallate. J. Food Sci..

[35] Kitagawa S., Nabekura T., Kamiyama S., Takahashi T., Nakamura Y., Kashiwada Y., Ikeshiro Y. (2005). Effects of alkyl gallates on P-glycoprotein function. Biochem. Pharmacol..

[36] Kitagawa S., Nabekura T., Nakamura Y., Takahashi T., Kashiwada Y. (2007). Inhibition of P-glycoprotein function by tannic acid and pentagalloylglucose. J. Pharm. Pharmacol..

[37] Kwon S.H., Kang M.J., Huh J.S., Ha K.W., Lee J.R., Lee S.K., Lee B.S., Han I.H., Lee M.S., Lee M.W. (2007). Comparison of oral bioavailability of genistein and genistin in rats. Int. J. Pharm..

[38] Eler G.J., Santos I.S., de Moraes A.G., Mito M.S., Comar J.F., Peralta R.M., Bracht A. (2013). Kinetics of the transformation of *n*-propyl gallate and structural analogs in the perfused rat liver. Toxicol. Appl. Pharmacol..

[39] Antonio L., Grillasca J.P., Taskinen J., Elovaara E., Burchell B., Piet M.H., Ethell B., Ouzzine M., Fournel-Gigleux S., Magdalou J. (2002). Characterization of catechol glucuronidation in rat liver. Drug Metab. Dispos..

[40] Antonio L., Xu J., Little J.M., Burchell B., Magdalou J., Radominska-Pandya A. (2003). Glucuronidation of catechols by human hepatic, gastric, and intestinal microsomal UDP-glucuronosyltransferases (UGT) and recombinant UGT1A6, UGT1A9, and UGT2B7. Arch. Biochem. Biophys..

[41] Yasuda T., Inaba A., Ohmori M., Endo T., Kubo S., Ohsawa K. (2000). Urinary metabolites of gallic acid in rats and their radical-scavenging effects on 1,1-diphenyl-2-picrylhydrazyl radical. J. Nat. Prod..

[42] Hu M., Chen J., Zhu Y., Dantzig A.H., Stratford R.E., Kuhfeld M.T. (1994). Mechanism and kinetics of transcellular transport of a new β-lactam antibiotic loracarbef across an intestinal epithelial membrane model system (Caco-2). Pharm. Res..

[43] Zhang L., Zheng Y., Chow M.S., Zuo Z. (2004). Investigation of intestinal absorption and disposition of green tea catechins by Caco-2 monolayer model. Int. J. Pharm..

[44] Niu T., Smith D.L., Yang Z., Gao S., Yin T., Jiang Z.H., You M., Gibbs R.A., Petrosino J.F., Hu M. (2013). Bioactivity and bioavailability of ginsenosides are dependent on the glycosidase activities of the A/J mouse intestinal microbiome defined by pyrosequencing. Pharm. Res..

[45] Yang Z., Gao S., Yin T., Kulkarni K.H., Teng Y., You M., Hu M. (2010). Biopharmaceutical and pharmacokinetic characterization of matrine as determined by a sensitive and robust UPLC–MS/MS method. J. Pharm. Biomed. Anal..

